# Teledentistry as a novel pathway to improve dental health in school children: a research protocol for a randomised controlled trial

**DOI:** 10.1186/s12903-019-0992-1

**Published:** 2020-01-14

**Authors:** Mohamed Estai, Yogesan Kanagasingam, Maryam Mehdizadeh, Janardhan Vignarajan, Richard Norman, Boyen Huang, Heiko Spallek, Michelle Irving, Amit Arora, Estie Kruger, Marc Tennant

**Affiliations:** 1 The Australian e-Health Research Centre, CSIRO, 147 Underwood Avenue, Floreat WA, Perth, 6014 Australia; 2 School of Human Sciences, University of Western Australia, Perth, Australia; 3 School of Dentistry and Health Sciences, Charles Sturt University, Orange, Australia; 4 Faculty of Medicine and Health, School of Dentistry, University of Sydney, Sydney, Australia; 5 School of Public Health, Curtin University, Perth, Australia; 6 Translational Health Research Institute, Western Sydney University, Campbelltown Campus, NSW Australia; 7 School of Health Sciences, Western Sydney University, Campbelltown Campus, NSW Australia; 8 Oral Health Services, Sydney Local Health District and Sydney Dental Hospital, NSW Health, Surry Hills, NSW Australia; 9 Discipline of Child and Adolescent Health, Sydney Medical School, Faculty of Medicine and Health, The University of Sydney, Westmead, NSW Australia

**Keywords:** Screening, Tooth decay, Dental photographs, School, Children

## Abstract

**Background:**

Despite great improvement in child oral health, some children subgroups still suffer from higher levels of dental caries. Geographic and socioeconomic barriers and the lack of access to dental care services are among common reasons for poor oral health in children. Historically in Australia, oral health therapists or dental therapists have been responsible for providing dental care for school children through the School Dental Services (SDS). The current SDS has been unable to provide sustainable dental care to all school children due to a reduction in workforce participation and limited resources. We propose a paradigm shift in the current service through the introduction of user-friendly technology to provide a foundation for sustainable dental care for school children.

**Methods/design:**

We describe an ongoing parallel, two-armed, non-inferiority randomised controlled trial that compares routine and teledental pathway of dental care in children aged 4–15 years (*n* = 250). Participating schools in Western Australia will be randomly assigned to the control or teledental group, approximately three schools in each group with a maximum of 45 children in each school. All participants will first receive a standard dental examination to identify those who require urgent referrals and then their teeth will be photographed using a smartphone camera. At the baseline, children in the control group will receive screening results and advice on the pathway of dental care based on the visual dental screening while children in the teledental group will receive screening results based on the assessment of dental images. At 9 months follow-up, all participants will undergo a final visual dental screening. The primary outcomes include decay experience and proportion of children become caries active. The secondary outcomes include the diagnostic performance of photographic dental assessment and costs comparison of two pathways of dental care.

**Discussion:**

The current project seeks to take advantage of mobile technology to acquire dental images from a child’s mouth at school settings and forwarding images electronically to an offsite dental practitioner to assess and prepare dental recommendations remotely. Such an approach will help to prioritise high-risk children and provide them with a quick treatment pathway and avoid unnecessary referrals or travel.

**Trial registration:**

Australian New Zealand Clinical Trials Registry, ACTRN12619001233112. Registered 06 September 2019.

## Background

Dental caries (tooth decay) remains the most prevalent chronic disease of childhood, even ahead of asthma and hay fever [1]. According to the Child Dental Health Survey 2010, the proportion of Australian children with caries experience (decayed, missing and filled teeth ‘dmft + DMFT’ > 0) varied from 48% for children aged 5 years to 69% for children aged 9 years [2]. There is a significant social gradient between socioeconomic status and the prevalence and severity of oral diseases which highlights oral health inequalities and inadequate funding for prevention and treatment [3]. Dental caries is an entirely preventable dynamic disease, with an interplay of risk factors that protect against or cause progression of the disease. In general diet, oral hygiene and fluoride exposure are the most common factors assessed when determining an individual’s risk level for the disease [4].

If dental caries remains untreated, it can have serious consequences that require costly treatment. Dental caries can lead to physical and psychosocial consequences including pain, sepsis, and disturbed eating, drinking and sleep pattern [3]. Poor oral health can have a significant impact on a child’s development including below average height, weight, and head circumference, and absenteeism from school [5, 6]. The demand for dental care is increasing and with it the total costs. Recurrent expenditure on dental care services in Australia increased from $6.6 billion AUD in 2006–07 to $10.2 billion AUD in 2016–17 (adjusted for inflation), at an average annual growth rate of 4.4% [7]. Dental extractions and restorations are the most common reported causes for hospital admission among Australian children [8]. For instance, between 2000 and 2009, a total of 43,937 children (0–14 years) were hospitalised due to various oral conditions in Western Australia (WA) with total costs of $92 million AUD [9].

Dental therapists or more recently oral health therapists (OHTs) have been responsible for providing most of the dental services (including examinations, fillings, extractions and preventive care) for school children ages 5–14 years through the government-funded school dental services (SDS) [10]. The history of SDS in Australia can be traced back to the 1920s, where it was designed to provide dental care for poor and disadvantaged children. Following the introduction of the Australian School Dental Scheme (ASDS) in 1973 [11], the SDS has expanded to cover all primary schools in all states and territories. Similar services are provided in several countries such as New Zealand, Canada, Hong Kong, Singapore and Malaysia [12]. Despite improvements in child oral health, nearly half the school children receiving dental care within the SDS do not enjoy good oral health [13]. In recent years, the SDS has suffered a reduction in workforce participation, scarcity of resources, and services remain largely concentrated in the metropolitan regions [14]. Until the 2000s, the employment of dental therapists was limited to the state-operated SDS [15]. Recently, many dental therapists have opted for private practice career or even leaving the profession which resulted in decreased retention and recruitment of dental therapists or OHTs in the SDS [16]. Therefore, the current SDS rarely provides sustainable dental care and is unable to meet all child oral health needs [13].

Children are at high risk of developing dental caries, but its impact can be reduced or prevented through early detection, early intervention, including caries risk assessment and tailored oral hygiene interventions and the provision of preventive dental care, particularly, in the early life [17, 18]. Therefore, it is important to regularly monitor the oral health of high-risk children to address the risk factors which can reduce the overall burden of the disease in the future [19]. However, reaching out to all school children, particularly, in rural regions, is challenging and costly. Thus, there is a need for an equitable and cost-efficient model of dental care to enhance the opportunity to provide sustainable dental care for school children, particularly those living in remote regions.

One of the potentially viable alternatives to address child oral health inequities and inequalities is the use of telehealth. Teledentistry is a form of telehealth that is specifically dedicated to dentistry and uses electronic medical records, information and communication technology (ICT), digital photography and the Internet for consultation, supervision or continuing education [20]. Telehealth, particularly, mobile technology, is an attractive innovation due to mobile connectivity, improving digital photography capabilities and the computational power of smartphones which allow their users to perform multiple tasks such as data processing, storage and transfer as well as access to low-cost, secure cloud storage. The use of a smartphone camera in dental photography can be useful for recording a baseline of oral health status pre-operatively, which can be used to confirm the diagnosis and preparing an appropriate treatment plan. As smartphones are widely carried and can be used at any time, they can be used as point-of-care devices to improve the delivery of patient-centred care. A telehealth model of care delivery suits role substitution in dentistry, particularly, facilitating data collection, health promotion and screening. At the screening level, trained teachers or school staff, using a user-friendly mobile technology, can help in collecting digital data from school children for later evaluation by a dental expert from a distance. Ample evidence indicated that the involvement of trained non-dental health personnel in the screening and provision of oral health promotion has been beneficial [21–23]. The use of telehealth can be ideal for the rural population who have no or limited access to health services [24].

The proposed project builds on previous validation trials that examined the feasibility of the teledentistry approach to dental screening [25–27]. The findings showed that the use of dental photography in dental screening is feasible and offered a moderate diagnostic accuracy relative to visual dental screening. Recent systematic reviews indicated that most reviewed studies concerned with teledental diagnosis demonstrated comparable results between photographic and visual inspection techniques [28, 29]. Although telehealth applications are becoming increasingly popular in dentistry, controlled assessments of clinical outcomes, long-term use and economic analyses of teledentistry are still limited [30–32]. A comparison of the costs of teledentistry and its alternatives is of great importance to those making future decisions about implementing a new service. In this study, we propose a paradigm shift in the existing dental services through role substitution and taking advantage of user-friendly mobile technology and the widely available Internet to improve school children access to dental services.

### Aims and hypotheses

#### Aims

The purpose of the proposed school-based research is to evaluate the efficacy and net economic benefits of a novel teledental pathway of dental care in improving the oral health outcomes of school children.

#### Objectives

- To determine the efficacy of a novel teledental pathway of dental care in improving oral health outcomes of school children relative to the usual pathway of dental care. *Hypothesis: The teledental pathway of dental care would be as effective as a routine pathway of dental care in improving children’s oral health.*
- To compare the costs of the teledental and usual pathway of dental care in school children, from a health system perspective. *Hypothesis: The teledental pathway of dental care would be cost-saving relative to the usual pathway of dental care.*
- To assess the diagnostic performance of dental screening on the basis of dental images relative to the reference visual dental inspection at the baseline. *Hypothesis: The photographic dental assessment will be as accurate and reliable as the standard visual dental screening.*

## Methods

This community-based project is a parallel, two-armed, non-inferiority randomised controlled trial (RCT) that seeks to determine the efficacy and net economic benefits of using telehealth to facilitate dental care delivery within school settings and aid in generating data which will assist to leverage the planning and justification for a multicentre large RCT. The study will be conducted in accordance with the SPIRIT Statement for reporting a clinical trial protocol [33] (checklist provided as Additional file 1).

### Web-based and mobile applications

**Fig. 1:**
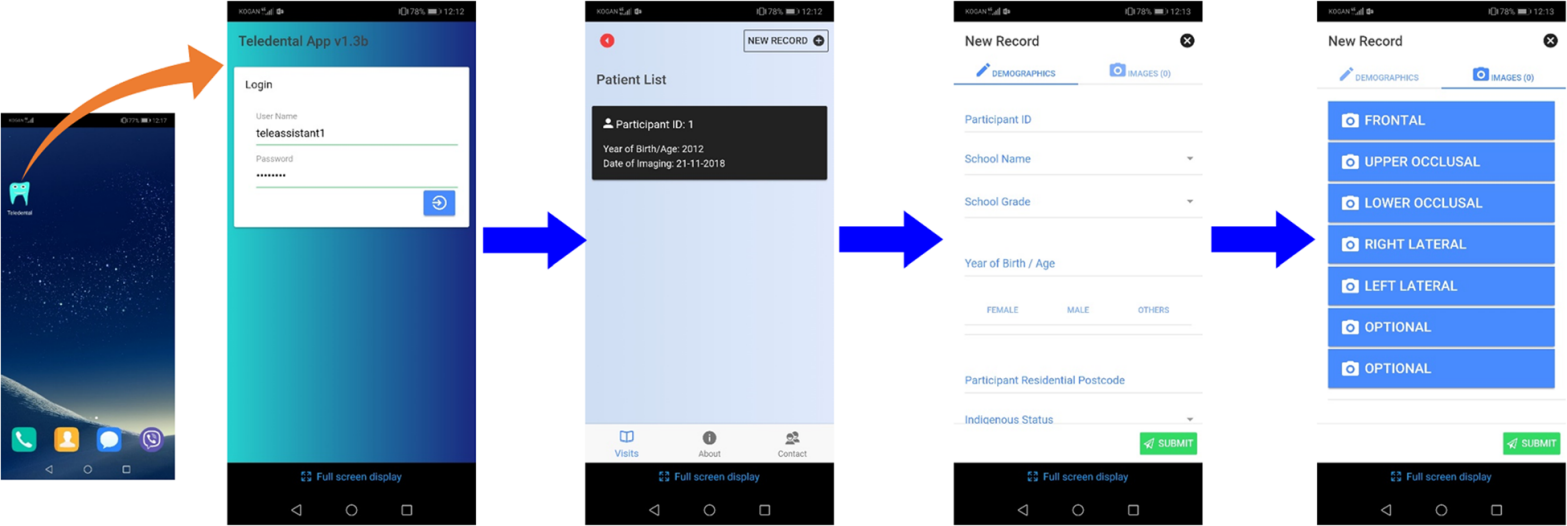
Teledental App used in dental photography

**Fig. 2:**
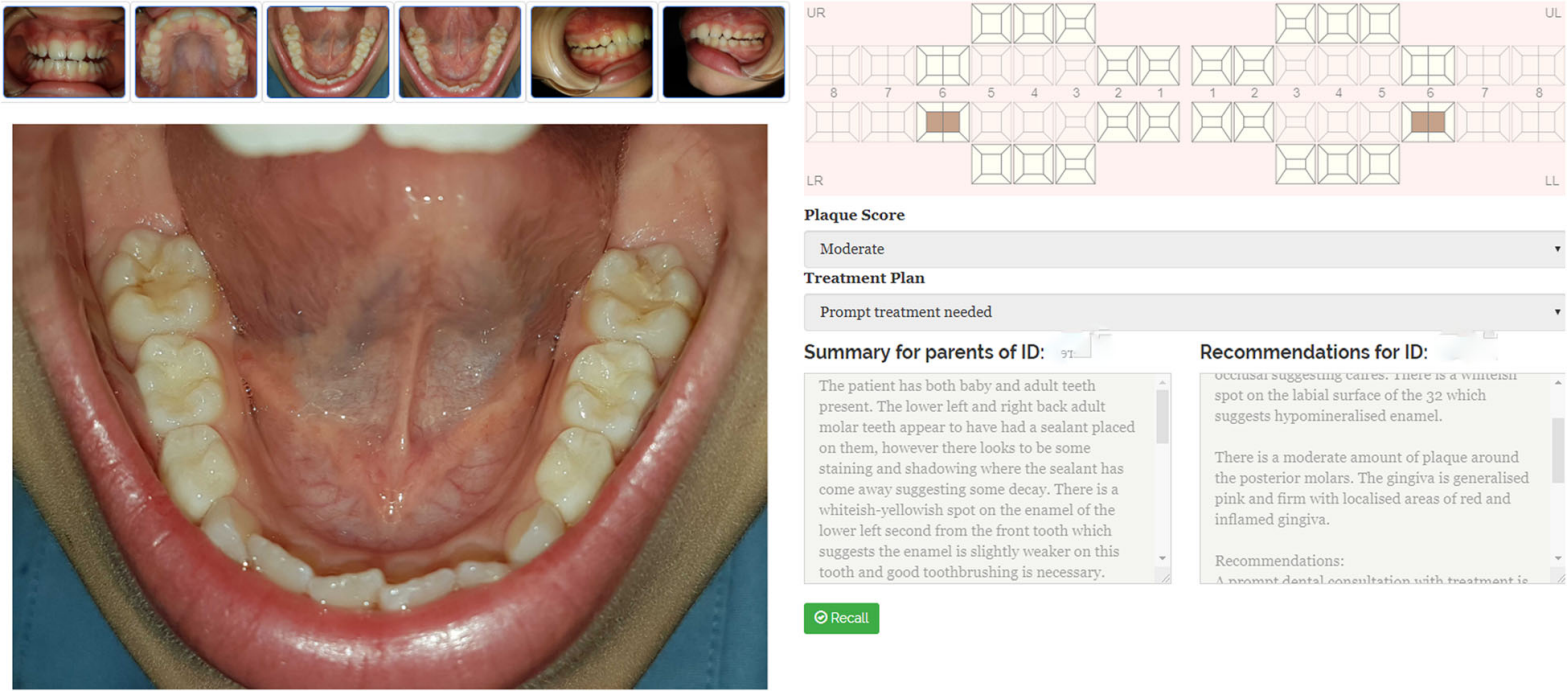
Screenshot of the Remote-I system depicting dentogram and dental images

Software engineers from the CSIRO’s Australian e-Health Research Centre (AEHRC), developed a reliable electronic health record system ‘Remote-I’ based on store-and-forward technology. There are two components of the prototype of the existing teledentistry system, (1) an image acquisition mobile App ‘Teledental’ to facilitate entry of patient information and capturing dental images, and then transferring the data to a secure drive using Wi-Fi or 4G network (Fig. 1). Capturing and reviewing images and lighting functions will be integrated into a single mobile App installed on a smartphone; and (2) web-based platform application ‘Remote-I’ for storage, viewing and charting of dental photographs (Fig. 2). The Remote-I system has been used in tele-retinal screening for diabetic retinopathy [34] and it was recently modified so it can be employed for dental screening purposes [25]. More information about the Remote-I and image acquisition mobile App are described in a previous study [25]. For this study, four Samsung® S7 devices with mobile Internet plan will be used in dental photography.

### Study setting

The study will take place in a school setting in Australia from 2018 to 2020. Participants will be recruited from schools in different geographical locations across WA.

### Participants’ recruitment

We will approach approximately 25 schools in WA, initially by email with a telephone follow-up, complying with good practice and research governance for undertaking studies within the education system. Parents of children aged 4–15 years, attending selected schools, will be invited to allow their children to take part in research using several advertising strategies including, schools’ newsletters, email lists and notice boards to help in achieving target sample. The inclusion criteria include children, aged 4–15 years, who are attending schools or college in WA in which the school principal has permitted for their school to be included in the trial will be included in the study, subject to their parents giving written consent. The exclusion criteria include children of the same age, in the same schools for whom parents have not given written permission for their children to take part. Once written permission is obtained from the schools, an information sheet and consent form will be mailed to parents of school children attending participating schools to invite them to participate. Besides, an information session will be scheduled for parents to provide more information about the project and respond to their enquiries.

### Allocation

**Fig. 3:**
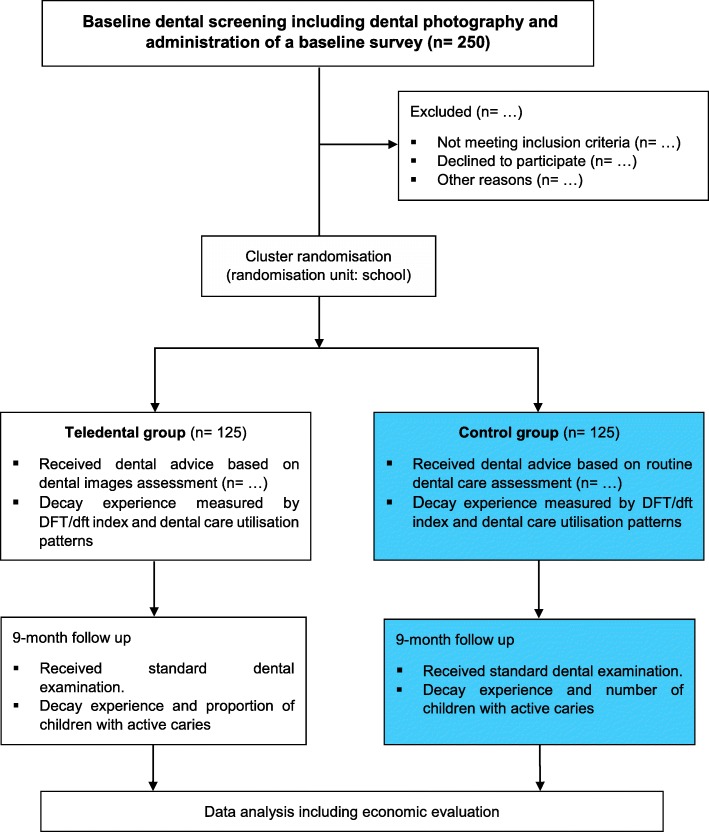
Flow diagram of the study

To avoid spill-over effects within a school, we will conduct randomisation using the school as the unit of allocation because the intervention is implemented at the level of the school (Fig. 3). The schools will be randomly allocated in a 1:1 ratio to one of the teledental or control group. For allocation of the participants, a computer-generated list of random numbers will be used. To approximate equality of sample sizes in the study groups, we will use block randomisation with computer-generated randomly permuted blocks of 4 at each school. School-specific randomisation lists are concealed by an independent researcher, and kept in sequence and opened as participants are examined at the baseline and found eligible for enrolment. The research team and data analysts will be blinded to group allocation and will not be aware of the randomisation procedure. Study participants (and their parents/guardians) and OHTs providing the intervention and/or collecting data cannot be blinded to group allocation.

### The intervention

At the baseline, the intervention includes dental assessment (through visual inspection or dental photography) and the provision of dental advice/recommendation that could include a referral to school dental providers, instructions or oral health promotion (OHP) leaflets, depending on the urgency and severity of the condition. The OHP leaflets are readily available on the Dental Health Services WA website (https://www.dental.wa.gov.au/) and permission has been obtained to use these leaflets.

#### Visual dental examination

A standard dental screening (without radiography) will be performed by registered OHTs (operators) who are blinded to the group allocation of the school. Head-mounted lighting, disposable mirrors and probes will be used to conduct the visual dental screening. Findings from the visual dental assessment will be recorded on an oral health assessment form that follows the guidelines for oral health surveys developed by the WHO [35]. Following the completion of the baseline dental screening, parents of school children allocated to the control group will receive a copy of the screening results based on the visual inspection performed by onsite OHTs. Based on the severity and urgency of dental conditions, children with active caries will be referred to local dental providers for further investigations. Children who are screened caries-free or at low-risk, will not receive active treatment other than OHP leaflets.

#### Photographic dental assessment

**Fig. 4:**
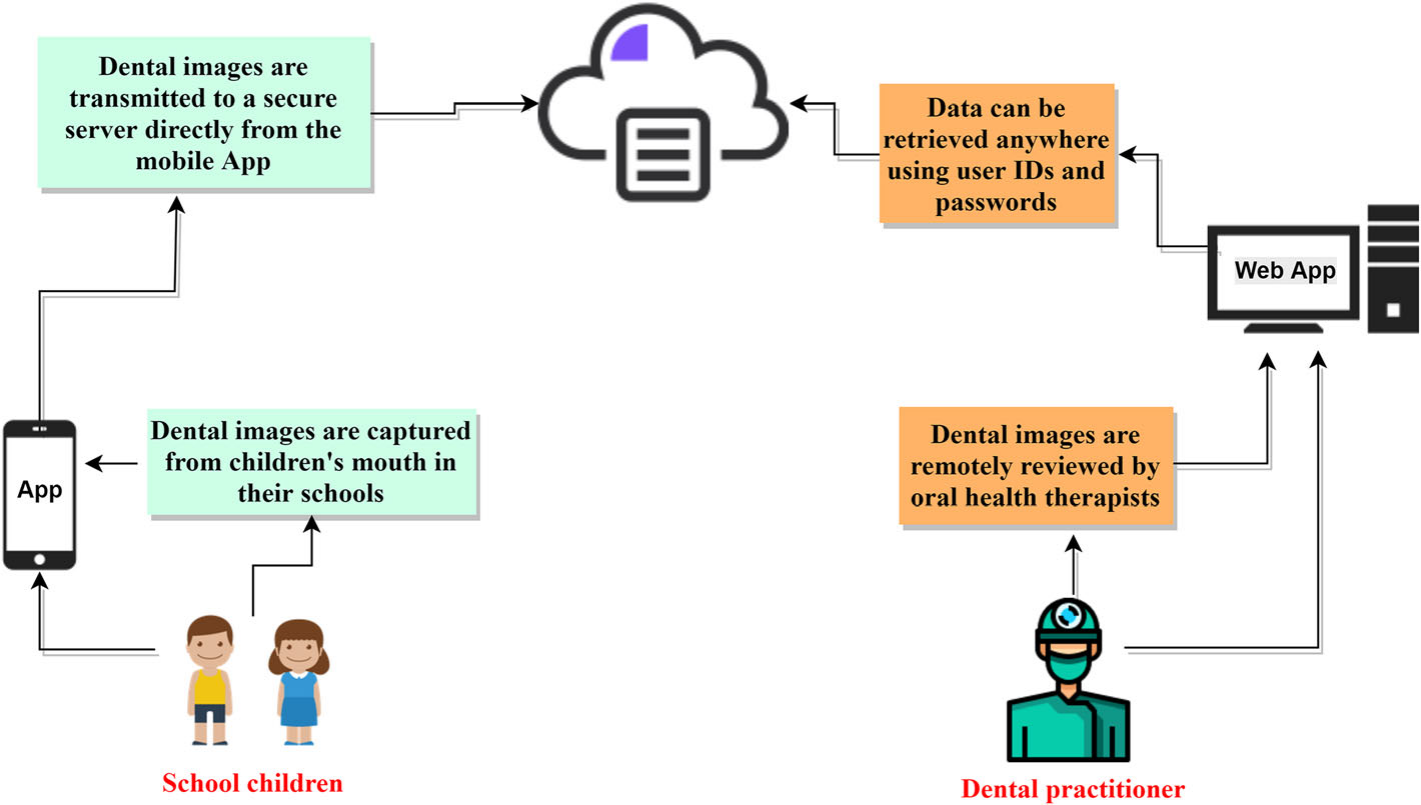
Main workflow of the teledental screening process

As per dental screening protocol used in previous trials [26, 27], tele-assistants (trained research assistants) will acquire still dental images from children using a novel image acquisition App installed in smartphone devices (Fig. 1). The tele-assistants will be provided with a dental photography protocol and will receive hands-on training on how to use the image acquisition App and capture good images. Disposable cheek retractors will be used to assist in viewing teeth during dental photography. Using the mobile App, the tele-assistants will first create a basic registration (includes a unique ID, age in years, gender and school name) for each participant. Then a minimum of five still dental images per child will be taken (front, right lateral, left lateral, upper occlusal, and lower occlusal views). Following the completion of dental photography and creating a record on the App, each participant’s set of data (including dental images and anonymous patient details) will be then directly uploaded from the mobile App to a secure drive located at the CSIRO, for later evaluation by charters (Fig. 4). The assessment of dental images will be performed independently by 2–4 registered OHTs (charters). Charters will receive instructions on how to log in and use the web-based application (Remote-I), review images and insert findings back to the system. After selecting a record from the database, a list of dental images and a predefined assessment chart appears for the charter to insert their findings. The chart includes a digital oral health assessment form, which aligns with the WHO protocol for oral health assessments [35]. The system enables charters to independently review dental images and submit reports or treatment plans to the Remote-I system (Fig. 2). The assessment of dental caries will be completed at the tooth level rather than the tooth surface, based on a protocol developed by the WHO [35]. A copy of the screening results will be mailed to the parents of children in the teledental group that includes advice on the pathway of dental care or OHP material based on the assessment of dental images .

#### Follow up

At 9 months following the baseline dental assessments, all participants will be invited to undergo a standard visual dental check-up to assess the dental status, determine if treatment was received and see whether parents adhere to the recommendations received at the baseline.

### Process evaluation

Regular auditing for OHTs will be conducted by an independent researcher to assess the intervention fidelity and determine whether the study protocol was followed as intended. Tele-assistants collecting dental images and OHTs participated in the charting of dental images will be surveyed to explore their acceptability of school dental screening based on dental photography.

To maximise retention of participants, all participants will receive a letter at the baseline on the importance of adhering to the intervention and on participation in the follow-up measurements as well as expected benefits including a free dental examination conducted within schools, and receiving screening report that includes preventive interventions to promote tooth brushing and healthy diet. To promote their active participation in the study, participants will receive Bright Smiles dental kits (consisting of toothbrush and toothpaste) at the end of the study.

### Outcome assessment

*Primary outcomes* include decay experience in either dentition as measured by the DFT/dft (decay, filled teeth) index at the baseline and 9-month follow up. Teeth will be classified as carious if have arrested caries, caries into dentine or enamel level caries. In addition to the proportion of children becomes caries-active at the 9-month follow-up which refers to the percentage of children developing one or more new caries in any of the previously caries-free teeth when assessed at the baseline. Data will be collected from one of our system’s functional components running on the backend to automatically record dental assessments based on dental images and through the oral health assessment forms completed by OHTs in the control group.

*Secondary outcomes* include the diagnostic performance of photographic dental assessment versus standard dental examination at the baseline. The participants’ dental care utilisation patterns collected via a baseline survey (Additional file 2) and adapted from Kopycka-Kedzierawski et al. [36]. Also, costs comparison between the two pathways of dental care will be conducted to identify the least costly alternative. The resources for the usual pathway of dental care will include vehicle costs, admin costs (e.g. telephone appointments, printing services and postage) and costs of consumables used in visual dental screening and the dental practitioner’s time. The resources for the teledental pathway of dental care include: software licence fees, ICT equipment, Internet and software engineer’s time, admin costs (e.g. telephone appointments, printing services and postage), costs of consumables used in dental photography, tele-assistants (photographer) time, and offsite dental practitioners’ time for reviewing dental images. Dental care costs for all children include the following: restorations, restorations with crowns and extractions. The costing data will be based on those incurred by the health system and will be derived from a survey and web-based open-access sources [37].

### Sample size calculation

The sample size calculation is based on a two-sided 95% CI for a single proportion using the Z-test approximation, an effect size of 0.1 and an expected observed proportion of 0.90. The number of participants with caries that meet the power of 0.8 is estimated to be 35 [n ≥ (Z^2^/m^2^) p (1 - p)]. With the prevalence of caries at 35% (1.86 × 65), 65 participants without caries are needed. After considering a 20% drop out rate, the proportion that gives the largest sample size shows that at least 125 children per group will be needed. Three schools will be required in each group with a maximum of 45 children in each school.

### Data analysis

The sensitivity and specificity values for the photographic caries assessment will be estimated and compared to the reference visual dental assessment at the baseline. Kappa statistic will be used to estimate the inter-rater reliability of the photographic dental assessments by two independent OHTs. 15% of dental images will be re-examined to test intra-rater reliability at least, 4 weeks after the initial review of images.

The baseline data will be used to generate descriptive statistics for the intervention and control groups (teledental versus usual care pathway). General linear regression models will be performed to compare the efficacy of the interventions on decay (mean DFT/dft) between intervention and control groups, adjusting for baseline data collected via a survey that includes tooth brushing (yes/no; frequency); dental visit patterns over the past year, child age, Indigenous status, Socio-Economic Indexes for Areas (SEIFA), private insurance status and educational level of parents. The measure of intervention efficacy will be the difference between the intervention and control groups in adjusted DFT/dft (caries incidence) per child. All analyses will be performed using SAS (version 9.4). In addition, a cost minimisation analysis will be undertaken to compare the costs associated with teledental versus the usual pathway of dental care over 9 months. This will include both the cost of the initial screening, and any subsequent interventions occurring over the 9 months. The analysis will be conducted from the perspective of the health system and, will not take into consideration the costs incurred by the families. All salaries used in the calculation will be based on Australian government rates and will include on-costs.

### Data management

Upon storage, all collected data will be stored de-identified in a secure institutional research data repository drive and/or locked filing cabinet. Data will only be accessible to authorised members of the research team. Data will be treated confidentially and stored for 10 years before being destroyed.

During data analysis, all data will be de-identified meaning identifiers will have been removed and replaced with a code. The master log linking the participant details with the unique study ID will be stored in an institutional password-protected drive.

#### Expected risks

Since no harmful consequences are expected from exposure to the intervention, a data monitoring committee is not needed. Ethics committees hold an annual review with the research team to monitor the research process. Unanticipated adverse events that are likely to be related to the trial will be recorded and reported to the relevant human research ethics committees.

#### Dissemination plan

No publication related to this study has been published or submitted to any journal. Findings will be reported according to the Consolidated Standards of Reporting Trials (CONSORT) Guidelines [38]. Research findings will be disseminated through reports, presentations, and scientific articles in peer-reviewed journals can be published in peer-review journals in a way that not leading to identifying participants. Authorship of the publications emerging from the study will be decided based on the guidelines of the International Committee of Medical Journal Editors. Main study protocol amendments will be reported when findings are disseminated.

## Discussion

The proposed study is innovative because it investigates the use of an equitable, low-cost, and mobile technology and the Internet to provide a foundation of equitable dental care for school children. The study addresses priorities raised in the National Oral Health Plan 2015–2024 [39]. More specifically, it identifies vulnerable and rural people as a priority area warranting actions to reduce inequity in dental care access.

Healthy behaviours and practices established in the early life of the child are often carried into adulthood [40]. Therefore, improving oral health in childhood is fundamental for reducing dental caries, early tooth loss and other oral diseases in adulthood and the advanced aged period. However, improving the population’s oral health is challenging, with an unequal distribution of the dental workforce and scarcity of resources making the transition to the prevention of dental diseases difficult. Many children, particularly those from advantaged backgrounds who attend for a routine check-up, are free of dental diseases and consuming most scarce resources. However, this is not the case for socially disadvantaged children who tend to have higher rates of dental diseases and much of it goes untreated [41]. Paradigm shifts in the current SDS whereby trained non-dental personnel, embracing a user-friendly mobile App, can work on the frontline to aid in identifying high-risk children. The front-line clinicians can collect oral health data (e.g. demographic information and still/live images) from screen-positive patients using their mobile devices and then store-and-forward the records to a dental expert at a distance to confirm the diagnosis or to request further investigation. Children who screened positive can then have a quick pathway to receive appropriate treatment while those who are at low-risk or caries-free will receive preventive care. This is important, as this could alleviate the anxiety of patients and if necessary, initiate earlier intervention. Also, this strategy could help direct specific dental care towards children that need it more and contributing to reducing inequalities in child oral health.

The expected benefits from the interventions are an improvement in the oral health status of school children, with a reduction in decay experience, disease prevention, and cost-saving due to a reduction of inappropriate referrals and unnecessary travel. Also, dental photography can be used to create digital dental archives which will help in assessing the progress of dental diseases and wide-reaching research opportunities. This study will provide an evidence-based framework for other research groups to carry out interventional RCTs to assess the effectiveness and cost-effectiveness of telehealth in improving care delivery and oral health in school children.

### Study status

The trial status of this study is currently collecting data. It is anticipated that data analysis will be completed in July 2020 with study completion achieved by October 2020.

## Supplementary information

**Additional file 1:** SPIRIT 2013 Checklist: Recommended items to address in a clinical trial protocol and related documents*

**Additional file 2:** Participant survey

## Data Availability

The datasets generated from the study cannot be publicly available as it requires research ethics approval and participants’ consents. A summary of final findings will be available for participants.

## Abbreviations

AEHRC: Australian e-Health Research Centre
CSIRO: Commonwealth Scientific and Industrial Research Organisation
DFT/dft: Count of decayed and filled teeth
ICT: Information and communication technology
OHP: Oral health promotion
OHT: Oral health therapist
SDS: School dental service
SEIFA: Socio-Economic Indexes for Areas
WA: Western Australia
WHO: World Health Organization
