# Supplementary material for: Teledentistry as a novel pathway to improve dental health in school children: a research protocol for a randomised controlled trial
Source: BMC Oral Health. 2020 Jan 14;20:11. doi: 10.1186/s12903-019-0992-1 (PMC6961289; doi:10.1186/s12903-019-0992-1)
Supplement: Supplementary file 2 — Additional file 2: Participant survey [file 12903_2019_992_MOESM2_ESM.docx]

# PARTICIPANT SURVEY

**Teledentistry as a Novel Pathway for Dental Care in Australian School Children**

**PARTICIPANT’S DEMOGRAPHIC INFORMATION**

|  |  |
| --- | --- |
| Participant ID |  |
| School name |  |
| Your child’s age (in years) |  |
| Indigenous status | Yes  No  Other |
|  |  |
| Your child’s gender | Male  Female  Other |
|  |  |
| Your residential postcode only |  |
|  |  |
| How often does your child usually brush their teeth each day? | None  1-2 times  More than 2 times |
|  |  |
| Does your child have private dental insurance? | Yes  No |
|  |  |
| What is your (mother) highest level of education? | Primary School  High School  Technical/College  University |

**DENTAL CARE SERVICE UTILISATION WITHIN THE PAST 12 MONTHS**

|  |  |
| --- | --- |
| Has your child visited a dental practice within the past year? | Yes  No |
|  |  |
| If no, what was the reason for delaying or avoiding a dental visit? | Cost  Distance to a dental practice  Others |
|  |  |
| If yes, what was the reason for the last dental visit? | Routine dental check-up  Dental problem  Others |
|  |  |
| What type of dental practice did your child attended? | Public clinic  Private clinic  School dental service |
|  | |
| How often does your child visit the dentist? | At least one visit a year  Visit but not every year  Do not visit at all |
|  |  |
| What type of dental service did your child receive within the past year? | Extraction  Filling  Scale/Clean  None of the above |
|  |  |
| Has your child been hospitalised for a dental problem within the past year? | Yes  No |
